# Blended diets and effects on gastrointestinal symptoms in children with gastrostomy tubes: A survey study

**DOI:** 10.1002/jpn3.70107

**Published:** 2025-06-09

**Authors:** Elena Scarpato, Linda Varcamonti, Silvia Salvatore, Claudio Romano, Francesca Marino, Maria Rosaria Serra, Massimo Martinelli, Erasmo Miele, Annamaria Staiano

**Affiliations:** ^1^ Department of Translational Medical Science, Section of Pediatrics University of Naples Federico II Naples Italy; ^2^ Pediatric Department, Hospital ‘F. Del Ponte’ University of Insubria Varese Italy; ^3^ Department of Human Pathology, Pediatric Gastroenterology and Cystic Fibrosis Unit University of Messina Messina Italy; ^4^ Department of Clinical Medicine and Surgery, Endocrinology Unit University of Naples Federico II Naples Italy

**Keywords:** clinical practice, enteral nutrition, feeding intolerance, home‐made feeds, intestinal motility

## Abstract

**Objectives:**

Interest is growing in the use of blended diets (BD) in children with gastrostomy. Evidence supporting the benefits of BD is conflicting, with limited data to assist physicians in clinical practice. The present survey aims to evaluate current use of BD in children and adolescents with gastrostomy.

**Methods:**

An online survey evaluating the use of BD in children with gastrostomy was sent to members of the European Society for Pediatric Gastroenterology, Hepatology and Nutrition with expertise on gastrointestinal motility. The questions assessed clinical indications, level of experience, preferred diet composition, and clinical outcomes.

**Results:**

We collected 26 questionnaires filled out by members from 13 different countries. Most of the respondents (84.6%) are pediatric gastroenterologists, with 69.2% visiting gastrostomy patients on a daily/weekly basis. The majority of the sample (61.5%) declares to use BD, but only in selected conditions, and only 38.5% reports an advanced experience with BD. The main reason for BD prescription is parental request (53.8%). In addition, 57.7% prefers homemade BD compared to only 15.4% that relies on commercial BD. Finally, 69.2% states to find an impact on gastrointestinal symptoms. Positive clinical outcomes are reported for vomiting (61.1%), constipation (50%), nausea (38.9%), and bloating (38.9%). The main reason for not using BD is the non‐standard nutritional composition.

**Conclusions:**

BD are commonly used in clinical practice. However, due to the lack of conclusive evidence and well‐designed studies, there is great variability in diet composition and clinical indications. Given the increasing demand from caregivers and the potential positive outcomes, further studies are needed to provide insights and guide healthcare professionals in their clinical practice.

## Abstract (highlights)

**What is Known:**

- Interest in the use of blended diets (BD) for gastrostomy‐fed children, as an alternative to commercial enteral nutrition formulas, is increasing.
- The use of BD may be beneficial for subjects experiencing gastrointestinal symptoms.

**What is New:**

- BD are frequently used in gastrostomy‐fed children, but there is wide variability in diet composition and clinical indications.
- Despite the increasing use of BD, most healthcare professionals report a low level of experience, highlighting the need for specialized training.

## 1 INTRODUCTION

Enteral nutrition (EN) support includes both the delivery of nutrients via feeding tubes or the provision of oral nutritional supplements, and is indicated in patients with at least a partial functioning of the digestive tract, when oral intake is insufficient to meet the patients' needs or is unsafe. Once the indication for EN has been formulated, the decision on the type of mixtures to be used requires careful evaluation, taking into account factors affecting both the patient and the mixture. Moreover, changing in the clinical conditions of the patient, progression of the pathology, and potential complications associated with EN may require reassessments of the nutritional protocol.^1^ Blended tube feeding is the provision of pureed foods administered via a gastrostomy tube.^2^ While aware of the benefits associated with the use of commercial formulas, such as body weight recovery and reduced microbiological risks compared to blended diets (BD), in recent years there has been a growing interest among families and caregivers in adopting home‐made foods as an alternative to commercial EN formulas.^3^ Family members perceive this choice, especially from an emotional perspective, as an opportunity to provide their child with the best possible care despite artificial nutrition. Another benefit associated with the use of BD in tube‐fed children is the improvement in gastrointestinal symptoms, such as retching and vomiting.

Gastrointestinal symptoms are prevalent in pediatric patients on long‐term enteral nutrition and, in selected conditions, anti‐reflux surgery may be required to address symptoms. An observational study evaluating the impact of BD in 33 children on EN highlighted that 73% of patients experienced at least a 50% improvement in gastrointestinal symptoms, including vomiting and retching.^4^ Another prospective study conducted on 20 children (aged 1–16 years) with a percutaneous endoscopic gastrostomy (PEG) that switched from a standard EN recipe to a home‐made BD based on natural foods confirmed those results, showing a reduction in gastrointestinal symptoms, including vomiting and gastroesophageal reflux disease (GERD) symptoms, and a decrease in the use of antiacid pharmacotherapy; however, an increase in the use of fecal softeners was reported.^5^ A position paper by the European Society for Pediatric Gastroenterology, Hepatology and Nutrition (ESPGHAN) Committee of Allied Health Professionals and Nutrition defined recommendations for the use of BD in tube‐fed children, highlighting that, despite the gold standard for EN is represented by the use of sterile and nutritionally complete commercial formulas, the use of BD may be beneficial in reducing gastrointestinal symptoms such as vomiting, constipation, and GERD and may potentially reduce medication dependency. However, the Authors conclude that published evidence on the potential health benefits and risks, and on the best way to use BD is scarce, leaving healthcare professionals in a relative knowledge vacuum on what to consider when caring for patients who wish to pursue this method of feeding.^6^

The aim of the present survey was to assess the actual clinical use of BD in children and adolescents with gastrostomy tubes, and to evaluate the impact of BD on gastrointestinal symptoms.

## 2 METHODS

### 2.1 Ethics statement

The survey included only general information and did not disclose sensitive patient data. For this reason, formal approval from the Ethical Committee was not sought.

### 2.2 Survey design

The questionnaire used in this study was developed, after a literature review, by three pediatric gastroenterologists (AS, ES, and SS) with expertise in the field. The survey was then pretested by a subgroup of healthcare professionals (three pediatric gastroenterologists and three dietitians) to evaluate the clarity and length of the questions, to estimate the time needed to complete the survey, and to improve the comprehension of the questions, identifying potential difficulties for the respondents.^7^

The questionnaire was created and distributed using the platform Google Forms (Google LCC, California, US), and was available for the responders from April to June 2022. The electronic survey consisted of 15 questions exploring: (a) respondent characteristics (location and field of practice, years of experience, frequency of caring for patients with gastrostomy); (b) use of BD in clinical practice, including level of experience, preferred composition, and delivery method; (c) impact of BD on gastrointestinal symptoms. The survey included mainly closed‐ended questions to ensure that time required to complete the form would not exceed 10 min.

### 2.3 Study population

The target population for this survey study consisted of the members of the ESPGHAN Neurogastroenterology, Motility and Functional Gastrointestinal Disorders Working Group, that includes healthcare professionals (general pediatricians, pediatric gastroenterologists, and allied health professionals) with specific interest in gastrointestinal motility disorders. The study population was invited to participate to the electronic survey on “The use of blended diets and their impact on gastrointestinal symptoms in children with gastrostomy tube” via an email sent to the Working Group mailing list. The email included the objectives of the survey and the link to access the questionnaire. A reminder was sent after 1 and 2 months from the first invitation. However, to avoid the risk of duplicate responses, a clear note requesting not to complete the survey more than once was included in the email. Participants to the survey were informed on the purpose and scope of the study, and participation was voluntary.

### 2.4 Statistical analysis

Descriptive statistics were used to describe the results. The findings for each question are reported as percentages, along with the number of respondents and the total respondents to the specific question (e.g., % (*n*/*N*)), as the number of respondents could vary in case of conditional questions. For opened questions, responses were categorized and analyzed narratively.

## 3 RESULTS

We collected 26 answers out of 82 invitations, with a response rate of 31.7%, from 13 different countries. Most respondents are pediatric gastroenterologists (22/26; 84.7%), working at University Hospitals (22/26; 84.7%), with more than 15 years of experience and visiting patients with a gastrostomy on a daily/weekly basis (18/26; 69.2%) (Table 1).

**Table 1:** Characteristics of the 26 respondents involved in the survey.

Characteristics	Overall *N* = 26
Field of practice (*n*; %)	
Pediatric Gastroenterology	22 (84.7)
Dietician	2 (7.7)
General Pediatrician	1 (3.8)
Dietetic Physician	1 (3.8)
Country of practice (*n*; %)	
Australia	2 (7.8%)
Belgium	2 (7.8%)
Brazil	1 (3.8%)
Croatia	1 (3.8%)
Greece	1 (3.8%)
Hungary	3 (11.5%)
Italy	8 (30.7%)
Lithuania	2 (7.8%)
Romania	1 (3.8%)
Serbia	1 (3.8%)
Slovenia	1 (3.8%)
The Netherlands	2 (7.8%)
United Kingdom	1 (3.8%)
Setting of practice	
University Hospital	22 (84.7)
First Level/General Hospital	3 (11.5)
Tertiary Hospital/National Institute	1 (3.8)
Years of experience	
>15 years	19 (73)
11–15 years	2 (7.8)
6–10 years	4 (15.4)
0–5 years	1 (3.8)
Frequency of visiting patients with gastrostomy	
Every day	7 (26.9)
Every week	11 (42.3)
Every month	5 (19.3)
Rarely	3 (11.5)

Regarding clinical practice, only 19.2% of the respondents (5/26) does not use BD for patients with gastrostomy, while most of the respondents (61.6%; 16/26) declares to use them, but only in selected conditions. The remaining 19.2% (5/26) uses BD in the majority of patients. However, despite their relatively wide use, only 38.5% (10/26) of the sample reports an advanced experience with BD, while 30.8% (8/26) declares to have an intermediate level of experience with need for supervision in clinical practice, and 30.8% (8/26) a low level of experience.

**Figure 1:**
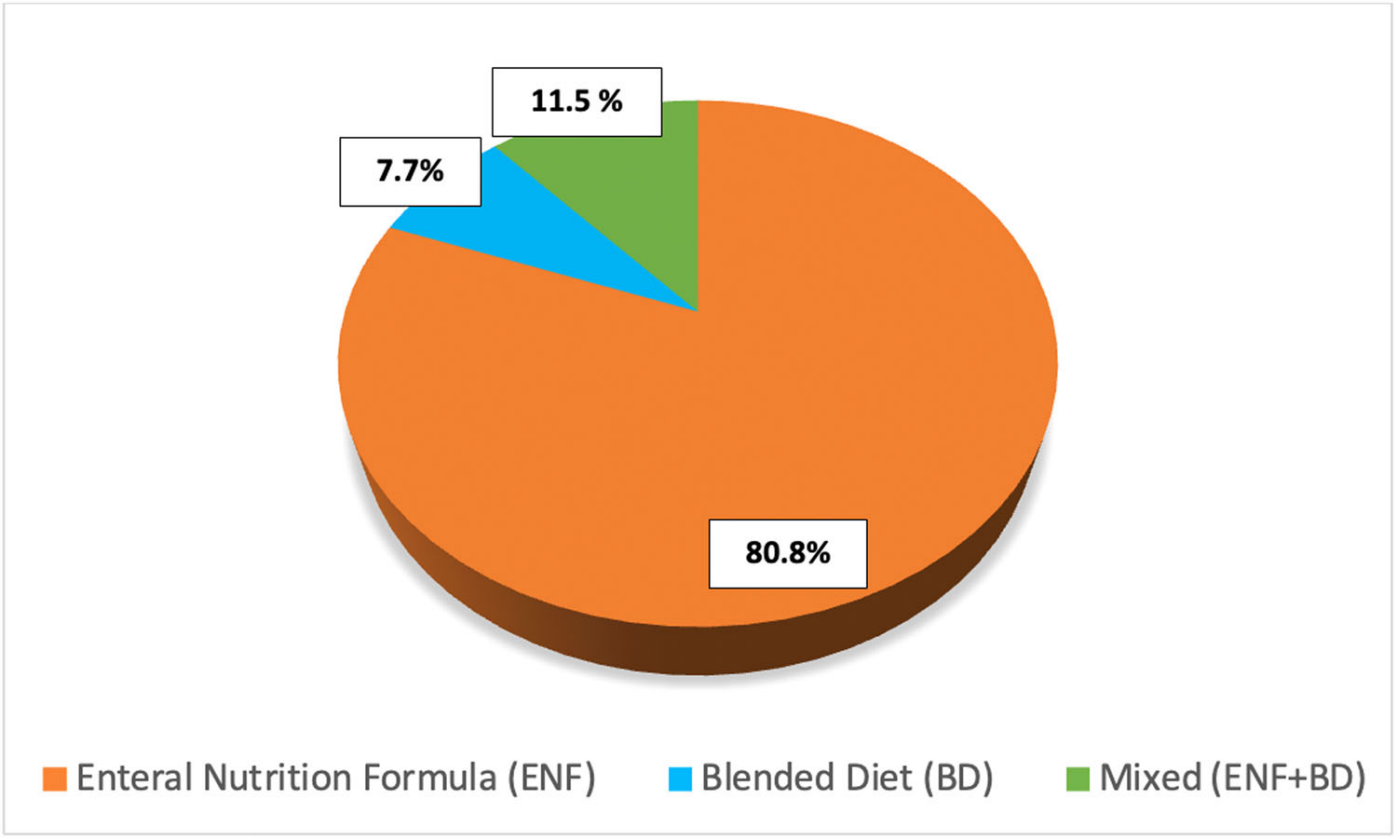
First choice for enteral nutrition in children with gastrostomy.

**Figure 2:**
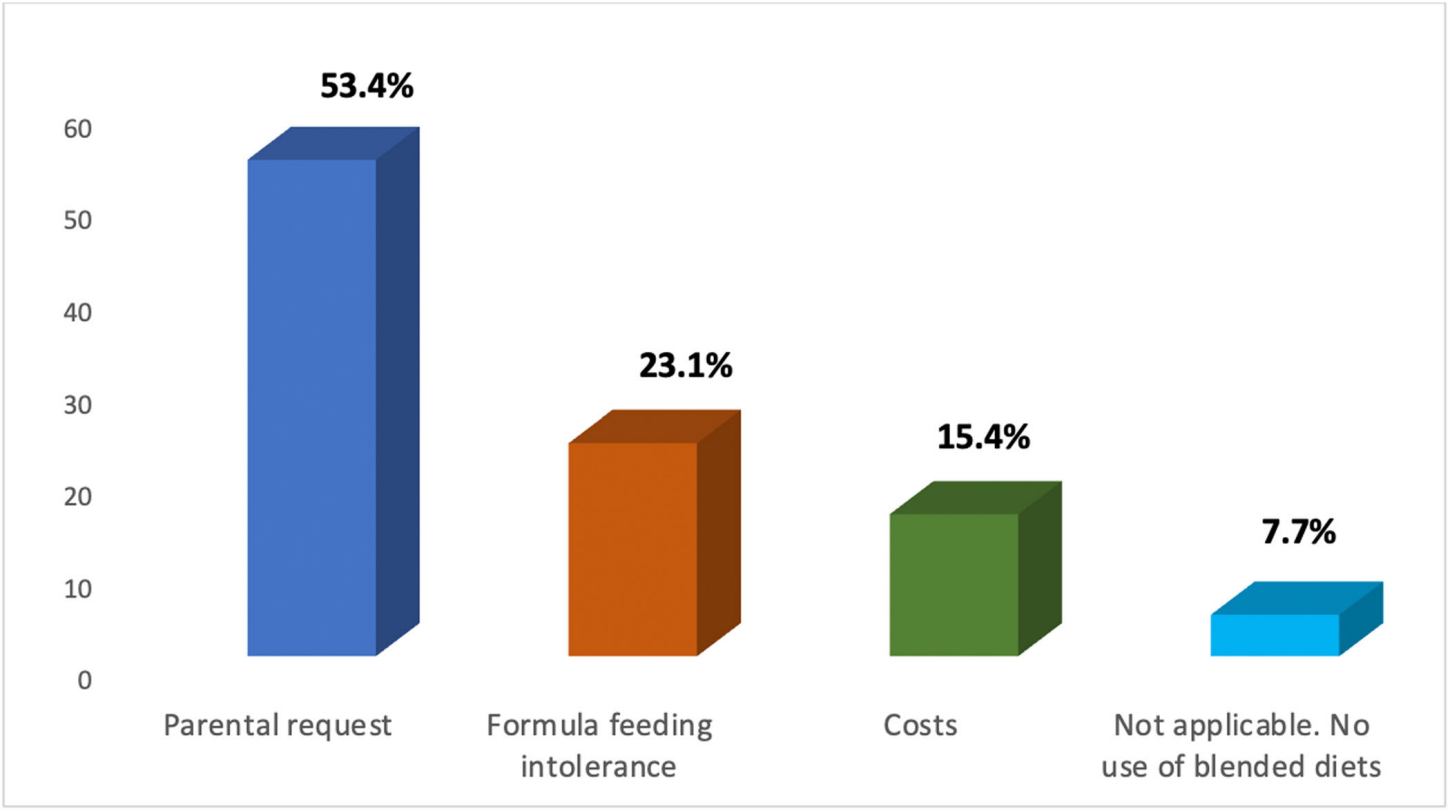
Main reasons for using blended diets in children with gastrostomy.

Most of respondents (80.8%; 21/26) consider commercial enteral formula the first choice for children with gastrostomy, while for 11.5% (3/26) of the sample, a mix of BD and commercial formula is preferred (Figure 1). The main reason for using BD is definitely parental request (14/26; 53.8%), followed by formula feeding intolerance (6/26; 23.1%) (Figure 2).

**Figure 3:**
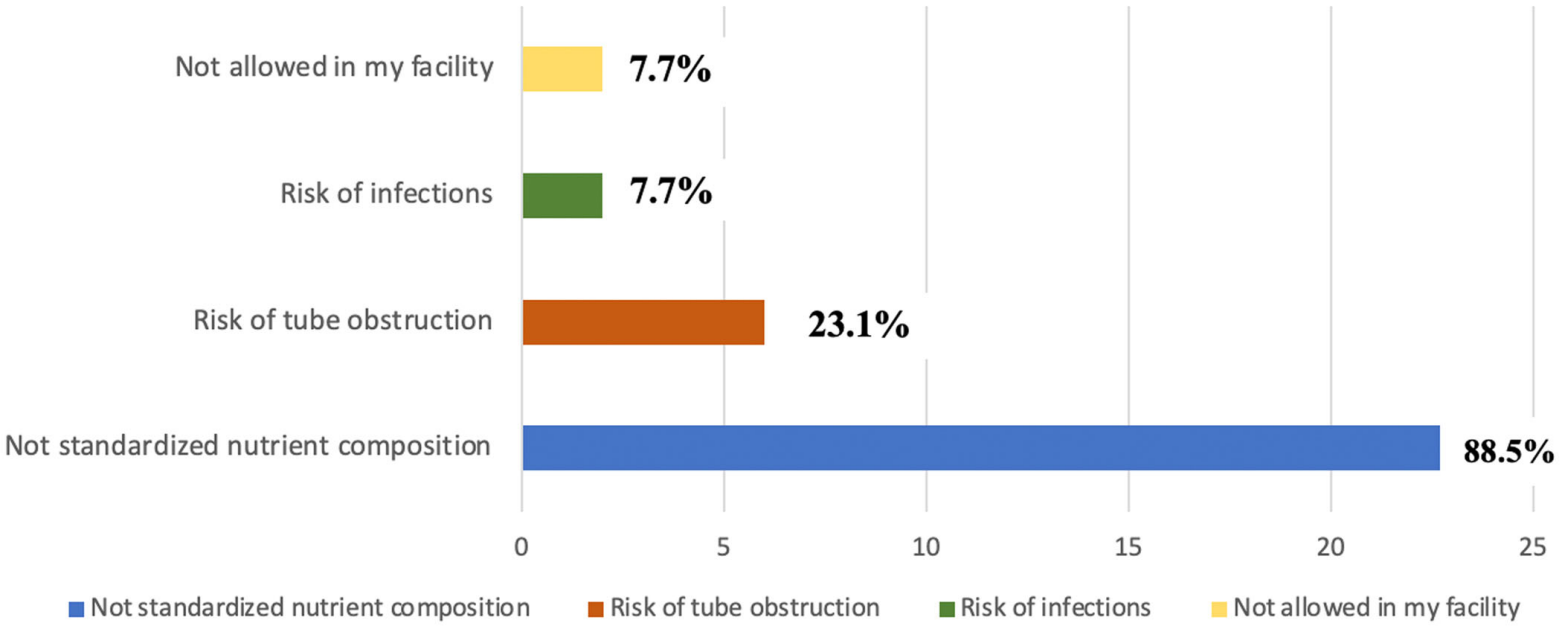
Reasons for not using blended diets in children with gastrostomy.

As for the preferred BD composition, there is a clear predominance of home‐made blended feeds (15/26; 57.7%), followed by commercial blended feeds (4/26; 15.3%), a combination of both (4/26; 15.3%), and adjustments based on parents' preferences (1/26; 3.8%). The remaining 7.8% (2/26) confirms not to use BD in clinical practice. The favorite delivery method for BD is the syringe (17/26; 65.4%); pumps are used by 23.1% (6/26), and gravity bags by 7.8% (2/26). The main reasons for not using BD are summarized in Figure 3. For this question more than one answer was possible.

Finally, we found that the general outcome of BD was positive for the majority of the sample (19/26; 73.1%). As for the impact of BD on gastrointestinal symptoms, 69.2% (18/26) declares to find an effect on gastrointestinal symptoms. In particular, a positive impact is reported on vomiting (11/18; 61.1%), constipation (9/18; 50%), nausea (7/18; 38.9%), and bloating (7/18; 38.9%). On the contrary, a negative impact is found on diarrhea (4/14; 28.6%), abdominal pain (4/14; 28.6%), vomiting (3/14; 21.4%), and bloating (3/14; 21.4%). For both those questions, more than one answer was possible. Finally, 65.4% (17/26) of the samples declares to be able to meet nutritional goals using BD.

## 4 DISCUSSION

In this survey study, we aimed to evaluate current indications for the use of BD and their impact on gastrointestinal symptoms, in children with gastrostomy. For this reason, we decided to target our survey to a subgroup of experienced healthcare professionals, members of the ESPGHAN Neurogastroenterology, Motility and Functional Gastrointestinal Disorders Working Group. The majority of our sample consists of pediatric gastroenterologists with more than 15 years of experience, working at University Hospitals, and regularly caring for children with gastrostomy, thus providing a reliable picture of the clinical management of this subgroup of patients.

Nutritional options for children with gastrostomies include commercial EN formulas, home‐made BD and ready‐to‐use commercial products based on real foods. Standard commercial EN formulas are designed to be used as a sole source of nutrition, providing all the required age‐appropriate essential macro‐ and micronutrients, and are still considered the gold standard for pediatric EN. However, considering both the reported benefits of BD, especially on gastrointestinal symptoms in long‐term tube‐fed neurologically impaired children, and the growing demand by parents, BD can be considered as a nutritional option, after a thorough risk assessment and evaluation of family's capability to provide BD at home.^6^ Our findings are in line with current recommendations, since for 80.8% of the participants standard commercial EN formula is still the preferred first choice for nutrition in tube‐fed children, while 61.5% declares to use BD, but only in selected conditions, mainly in case of parental request or intolerance to standard commercial EN formula. Of note, 30% of our respondents declares to deliver BD via pump/gravity bags. Nevertheless, the use of pumps/gravity bags to deliver boluses can cause inaccurate rate of delivery or clogging, due to variability in viscosity. For this reason, thinner viscosity is needed in case of use of an automated feeding pump or a gravity bag.^8^

The hesitancy of healthcare professionals to endorse BD use could be partly due to the lack of robust evidence on their nutritional adequacy and safety.^9^, ^10^ In fact, according to our data one of the main reasons for not using BD is the non‐standardized nutrient composition. A rapid review examining the use of BD in enterally fed children demonstrated wide variability in the nutritional content of BD in different hospital settings,^11^ and this concern seems also in line with the findings from a study by Chandrasekar et al.^12^ conducted on 41 gastrostomy‐fed children receiving either a BD or a commercial EN formula. The authors found that children fed BD had lower weight and BMI z‐scores and higher rates of malnutrition, compared to children fed commercial EN formula, suggesting that BD alone may not be sufficient to support adequate growth. For this reason, a mix of BD and commercial EN formula could represent a valid option, as chosen by 11.6% of our respondents.^11^

Additional reasons for not using BD are the known risks of tube obstruction and infections, secondary to microbial contamination. In fact, improper food hygiene could potentially heighten the risk of gastrointestinal infections in subjects fed BD. In a study conducted on adults, Vieira et al.^13^ evaluated microbiological quality of enteral feeds and found that home‐made BD had higher levels of bacterial contamination compared to commercial EN formula. Nevertheless, in a recent cohort study involving 180 gastrostomy fed children (104 home‐made BD and 76 EN formula) no evidence of an increase in the number of gut infections, stomia site infections, and pneumonia was found in the home‐made BD group compared to the EN formula group.^14^ The risk of complications can be minimized with adequate education and training of families to the use of BD.

An additional factor for not using BD could be self‐confidence in the management of this nutritional strategy. Notably, although our sample includes mainly highly experienced Pediatric Gastroenterologists, only a minority of the respondents (38.5%) states to have an advanced level of experience with the use of BD, while most of the sample (61.6%) declares to have a low level of experience or to need support/supervision for the use of BD in clinical practice. In fact, as already reported by Eustace et al., increasing interest on BD does not parallel with physicians' familiarity with this nutritional option, highlighting the need for specific education of healthcare professionals caring for enterally fed patients.^15^ The Authors detected an overall poor level of confidence with BD among physicians and advanced practice providers, probably due to the lack of previous training or guidance on BD, with 73.3% of the sample feeling either not very confident or not confident at all, in line with data from our sample. A useful support for healthcare professionals working in the field of home enteral tube‐feeding is represented by practice toolkits defined by scientific associations, that provide basic background to BD use and best practice guidance on nutritional adequacy, food preparation, administration of feeds, and clinical monitoring.^8^, ^16^

Data from our sample show that the main reasons for using BD are parental request and formula feeding intolerance, in line with an investigation involving Greek Registered Dietitians that identified as primary reasons for BD prescription parental request (72%) and improvement of food tolerance (22.9%).^17^ Regarding parental request, the adoption of a home‐made BD can enhance caregivers' satisfaction. As reported by Hron et al.,^18^ parents who administer a BD feel gratified by the alignment of their child's nutritional regimen with familial philosophy, and by the reduced medicalization of daily routine. As for feeding intolerance management, several studies report that patients unable to tolerate standard commercial EN formula may benefit from BD use.^19^, ^20^, ^21^ Specifically, a significant improvement in constipation, nausea/vomiting, and reflux with reduced need of anti‐reflux medications is reported in children fed BD compared to those receiving standard commercial EN.^12^ Data from our sample are consistent with these results, as most of the respondents report a positive effect on vomiting, constipation and nausea.

Overall, we believe our results are useful to clarify current use of BD by healthcare professionals involved in the care of children by gastrostomy tubes.

This survey has some limitations. First, although the survey was designed by three pediatric gastroenterologists with expertise in the topic, the questionnaire was neither validated nor tested for reliability. Nevertheless, the study has been piloted by a subgroup of healthcare professionals including both pediatric gastroenterologists and dietitians, to evaluate the clarity of the questions and improve the comprehension of the survey. The response rate appears to be relatively low (31.7%). However, for survey‐based studies, a response rate of 40% is typically acceptable, and it is known that online surveys generally have poorer response rates compared to postal surveys.^22^ For this reason, we are confident that our response rate reaches an acceptable threshold to reduce nonresponder bias. Finally, since the survey aimed to explore the impact of BD on gastrointestinal symptoms, it has been sent only to the members of the ESPGHAN Motility Working Group; however, sending the survey to all ESPGHAN members would have provided even more representative results.

## 5 CONCLUSIONS

Our survey shows that experienced pediatric gastroenterologists consider BD as a valid option for the nutritional management of tube‐fed children, despite the lack of detailed, dedicated, evidence‐based guidelines. The impact on gastrointestinal symptoms is considered positive. However, we found variability in diet composition and clinical indications, likely due to the scarcity of conclusive evidence and well‐designed original studies.

Given the increasing demand from caregivers, and the potential positive impact on enteral feeding tolerance and gastrointestinal symptoms, the need for specialized training of healthcare professionals on the use of BD appears particularly relevant.

## CONFLICT OF INTEREST STATEMENT

The authors declare no conflict of interest.
